# Evaluation of the New Robotic Platform “HINOTORI™” in Urologic Robot-Assisted Surgery: From a Comparison with da Vinci^®^ Surgical System in Sacrocolpopexy

**DOI:** 10.3390/jcm14092954

**Published:** 2025-04-24

**Authors:** Tetsuya Fukumoto, Takatora Sawada, Keigo Nishida, Tomoya Onishi, Ryuta Watanabe, Kenichi Nishimura, Noriyoshi Miura, Yuki Miyauchi, Tadahiko Kikugawa, Takashi Saika

**Affiliations:** Department of Urology, Ehime University Graduate School of Medicine, Toon 791-0295, Japan; sawada.takatora.dx@ehime-u.ac.jp (T.S.); nishida.keigo.ir@ehime-u.ac.jp (K.N.); onishi.tomoya.ey@ehime-u.ac.jp (T.O.); watanabe.ryuta.cu@ehime-u.ac.jp (R.W.); nishimura.kenichi.vx@ehime-u.ac.jp (K.N.); miura.noriyoshi.mk@ehime-u.ac.jp (N.M.); miyauchi.yuki.mf@ehime-u.ac.jp (Y.M.); kikugawa.tadahiko.my@ehime-u.ac.jp (T.K.); saika.takashi.ol@ehime-u.ac.jp (T.S.)

**Keywords:** HINOTORI™, robot-assisted surgery, sacrocolpopexy

## Abstract

**Background/Objectives:** HINOTORI™ is a robotic-assisted surgical platform developed in Japan. It has been applied in urologic procedures such as robot-assisted radical prostatectomy (RARP) and partial nephrectomy (RAPN). This study aimed to evaluate the clinical performance of HINOTORI™ compared with the da Vinci^®^ surgical system by analyzing outcomes of robot-assisted sacrocolpopexy (RSC) performed by a single skilled surgeon using a uniform surgical procedure. **Methods:** A total of 125 patients who underwent RSC for pelvic organ prolapse (POP) were analyzed. Surgical outcomes were compared between the HINOTORI™ (h-RSC group) and da Vinci^®^ (d-RSC group) platforms. Evaluated parameters included operative time, robotic console time, anterior compartment dissection time, suture time per stitch, perioperative complications, hospital stay, and POP recurrence. **Results:** Operative and robotic console times were significantly longer in the h-RSC group (148 vs. 139 min, *p* < 0.005; 109 vs. 95 min, *p* < 0.001). Anterior compartment dissection time showed no significant difference (*p* = 0.58), but suture time per stitch was longer in the h-RSC group (76 vs. 60 s, *p* < 0.005), possibly due to limited suture-cutting functionality, requiring manual assistance. No significant differences were observed in perioperative complications, hospital stay, and POP recurrence. **Conclusions:** HINOTORI™ demonstrated surgical precision and safety comparable to the da Vinci^®^ surgical system. It may serve as a viable alternative robotic platform, supporting broader adoption of robot-assisted surgical technologies.

## 1. Introduction

The advent of robot-assisted surgical systems has revolutionized various surgical specialties, including urology, general surgery, thoracic surgery, gynecology, otolaryngology, and cardiac surgery. Among these, the da Vinci^®^ system (Intuitive Surgical, Sunnyvale, CA, USA) has emerged as the most widely adopted and well-established platform for robot-assisted surgery [1]. Recently, various global companies have introduced some new robotic platforms. Of these, the HINOTORI™ surgical system (Medicaroid Corporation, Kobe, Japan), the first Japanese robot-assisted platform, received regulatory approval in August 2020. The HINOTORI™ surgical system may offer an advantage due to its docking-free design. This feature allows the robotic arms to operate without direct trocar fixation, thereby minimizing excessive stress on the abdominal wall. Additionally, the system’s features are designed to enhance surgical precision, procedural adaptability, and overall clinical efficacy in robot-assisted surgery and may offer performance comparable to existing platforms [2]. To objectively evaluate the performance of this new platform, we selected robot-assisted sacrocolpopexy (RSC) for pelvic organ prolapse (POP) as the model procedure. RSC requires multiple technically demanding steps—including precise tissue dissection, intracorporeal suturing, and optimal surgical field exposure—that are directly influenced by the functional capabilities of robotic systems. In this study, all procedures were performed by a single experienced surgeon using a standardized technique, thereby minimizing procedural variability and enabling a clearer comparison between platforms. POP refers to the descent of pelvic organs due to the weakening of the vaginal and pelvic floor support structures. Clinical manifestations include vaginal bulging, pelvic pressure, and lower urinary tract, bowel, or sexual dysfunction. Diagnosis is based on physical examination, typically revealing anterior vaginal wall prolapse or apical descent. The pelvic floor musculature, ligaments, and connective tissue play essential roles in organ support, continence, and overall pelvic stability [3]. The reported prevalence of POP ranges widely from 3% to 50%, depending on the population studied [4,5,6]. Multiple risk factors have been associated with POP, including genetic predisposition, parity, mode of delivery, prior hysterectomy, menopause, and altered estrogen receptor distribution [7]. Theoretical models such as Petros’ Integral Theory and DeLancey’s Levels of Support have been widely used for diagnostic and classification purposes [8]. While mild or asymptomatic POP can often be managed conservatively, symptomatic POP frequently requires surgical intervention. Given the high prevalence of lower urinary tract symptoms (LUTS)—with up to 88% of symptomatic patients presenting with cystocele—urologists are increasingly involved in the diagnosis and treatment of pelvic floor disorders [4]. Surgical options include native tissue repair or the use of polypropylene mesh augmentation, with robot-assisted sacrocolpopexy using dual mesh placement now regarded as the gold standard for apical prolapse repair [9]. Because RSC tasks, such as dissection and suturing, are highly dependent on robotic functionality, this procedure is ideal for comparing robotic systems. In contrast, procedures like prostatectomy or nephrectomy involve greater variability in organ size and complex vascular anatomy, which could confound platform-based performance assessments.

## 2. Materials and Methods

### 2.1. Patients

From January 2019 to March 2024, a total of 172 patients underwent sacrocolpopexy at the Department of Urology, Ehime University Hospital. All procedures were performed using robot-assisted surgery. Among them, 125 patients were operated on by a single surgeon, and these cases were included in the study. Thirty patients who underwent sacrocolpopexy using the HINOTORITM surgical system were assigned to the h-RSC group, while 95 patients undergoing the procedure with the da Vinci^®^ surgical system were classified into the d-RSC group. All procedures were performed by a single surgeon to ensure uniformity in surgical technique and minimize bias in the comparison between the two robotic platforms. Perioperative data and clinical outcomes of the patients who were retrospectively evaluated.

### 2.2. Patient Positioning and Port Placement

All procedures were performed with the patient in the lithotomy position under a 15–20° Trendelenburg tilt. The pneumoperitoneum was established and maintained at a pressure of 8–10 mmHg. A total of five trocars were utilized. A 12-mm camera port was inserted at the umbilicus. Three 8-mm robotic instrument ports were placed approximately 7–8 cm apart: one to the right of the camera port (for fenestrated bipolar forceps), one to the left (for monopolar curved scissors and a needle driver), and one lateral to the right-sided port (for grasping forceps). An additional 12-mm assistant port was inserted in the far-right lower quadrant.

### 2.3. Surgical Procedure

#### 2.3.1. The Procedure Proceeded as Follows

First, physiological adhesions of the sigmoid colon were dissected, and the colon was straightened to improve visualization and create adequate working space within the pelvic cavity. Dissection was then carried out between the bladder and anterior vaginal wall, down to the level of the bladder neck. The anterior mesh was sutured to this plane. A supracervical hysterectomy was subsequently performed, followed by fixation of the anterior mesh to the cervical stump. Dissection was then extended to the posterior compartment, developing the space between the rectum and posterior vaginal wall, to which the posterior mesh was affixed. Following exposure of the sacral promontory, the arms of both anterior and posterior meshes were anchored to the anterior longitudinal ligament under appropriate tension. Finally, the peritoneum was closed over the mesh, and an anti-adhesion barrier was applied to complete the procedure.

#### 2.3.2. Surgical Parameters and Clinical Outcomes

Perioperative outcomes, including operative time, robotic console time, and complication rates, were assessed. To compare differences between the surgical platforms, operative time for anterior compartment dissection and suturing was analyzed. Anterior compartment dissection was defined as dissection extending distally to either the bladder trigone or the bladder neck. Since the number of sutures varied across cases, suturing time was standardized by calculating the time per suture.

Postoperative outcomes, such as length of hospital stay, de novo stress urinary incontinence (SUI), mesh exposure, and POP recurrence, were also evaluated. Due to the differing initiation periods of each robotic platform, analysis of postoperative outcomes was limited to cases with a follow-up duration of at least six months. Complications were classified according to the Clavien–Dindo classification system, and POP recurrence was defined as a postoperative POP-Q stage of II or higher.

Subjective symptoms were assessed using the Pelvic Floor Distress Inventory (PFDI-20) [10,11]. Additionally, the PFDI-20 was used to evaluate the presence and impact of pelvic floor disorders. The PFDI-20 is a validated instrument previously applied in populations comparable to ours. It comprises 20 items divided into three subscales, each evaluating different aspects of pelvic floor dysfunction: Pelvic organ prolapse symptoms (POPDI-6 subscale) (questions 1–6), Colorectal-anal symptoms (CRADI-8 subscale) (questions 7–14), Urinary symptoms (UDI-6 subscale) (questions 15–20). Each question follows a 0–4 response format, categorizing dysfunction into four levels: none, mild, moderate, and severe. Each subscale ranges from 0 to 100, representing minimal to severe dysfunction. The total PFDI-20 score is the sum of the three subscales, with a maximum score of 300.

#### 2.3.3. Statistical Analyses

Results were presented as the median, interquartile range (IQR), and range. Statistical comparisons between groups were performed using the Mann-Whitney U-test, with differences considered statistically significant at *p* < 0.05. All statistical analyses were conducted using EZR, a modified version of the R commander designed to incorporate statistical functions frequently used in biostatistics [12].

## 3. Results

A total of 125 patients with POP underwent RSC (h-RSC group: 30 patients, d-RSC group: 95 patients). The baseline characteristics of the h-RSC and d-RSC groups are summarized in Table 1. There were no statistically significant differences between the two groups in terms of age, median body mass index (BMI), parity, POP-Q stage, previous hysterectomy, or concomitant ventral rectopexy.

The perioperative outcomes are presented in Table 1. The operative time (min) was longer in the h-RSC group than in the d-RSC group (median 148 min [range, 121–200] vs. median 139 min [range, 98–215]; *p* < 0.005). Similarly, robotic console time (min) was longer in the h-RSC group than in the d-RSC group (median 109 min [range, 84–160] vs. median 95 min [range, 59–156]; *p* < 0.001).

**Figure 1:**
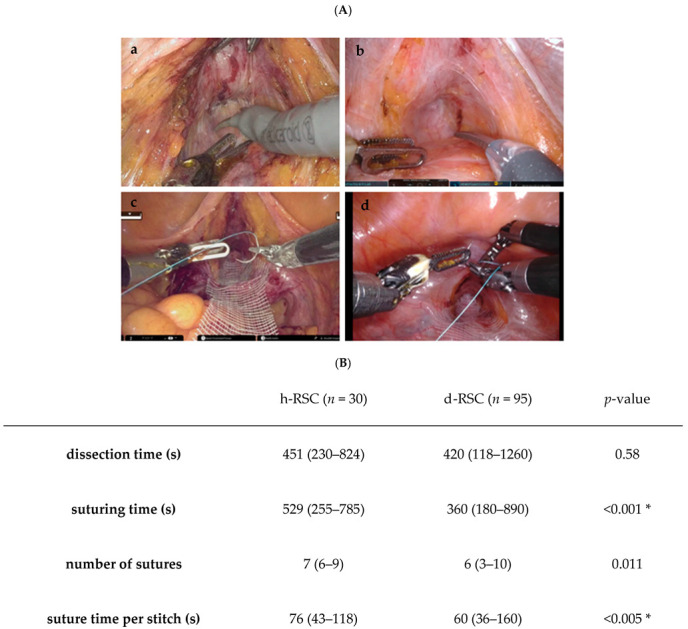

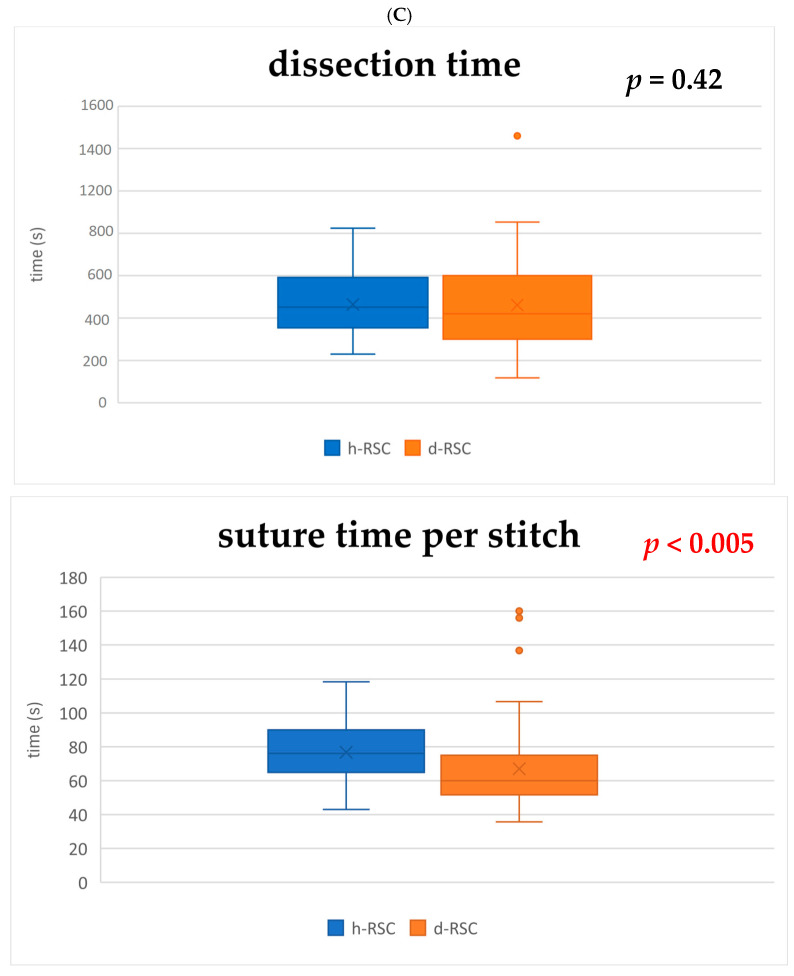
Anterior compartment dissection and sutures. (**A**) An intraoperative video demonstrating the technique is provided. (**a**,**c**): HINOTORI™-RSC. (**b**,**d**): da Vinci^®^-RSC. (**B**) A comparison table of the HINOTORI™ and da Vinci^®^ surgical systems. * Statistically significant. (**C**) A box plot was generated based on the data presented in (**B**).

A subgroup analysis of the anterior compartment dissection time (s) showed no statistically significant difference between the groups (h-RSC group: median 451 s [range, 230–824] vs. d-RSC group: median 420 s [range, 118–1260], *p* = 0.58). However, the suture time per stitch (s) was significantly longer in the h-RSC group than in the d-RSC group (median 76 s [range, 43–118] vs. median 60 s [range, 36–160], *p* < 0.005) (Figure 1). The longer stitching time in the h-RSC group may be attributed to differences in EndoWrist instrument functionality, particularly in suture-cutting mechanisms, which required manual assistance in the h-RSC group.

Postoperative outcomes in patients with at least six months of follow-up are presented in Table 2. Although there was a difference in observation periods between the groups (*p* < 0.001), no statistically significant differences were observed in the length of hospital stay (*p* = 0.67), mid-urethral sling surgery (*p* = 0.41), postoperative complications (*p* = 0.48), or POP recurrence rates (*p* = 0.55). Both surgical approaches demonstrated comparable safety and efficacy profiles, suggesting that the two robotic platforms are clinically equivalent in terms of safety and efficacy.

Additionally, Table 3 presents the Pelvic Floor Distress Inventory (PFDI-20) scores at preoperative and 1-month postoperative time points. Both groups demonstrated significant improvements in PFDI-20 scores postoperatively (*p* < 0.001). However, in the h-RSC group, no statistically significant difference was observed in the Colorectal-anal symptoms (CRADI-8) subscale between the pre- and postoperative periods, which may be attributed to the small sample size.

## 4. Discussion

The evolution of master-slave robotic platforms has played a pivotal role in advancing robot-assisted surgical systems since their first application in human surgery. Since receiving regulatory approval in 2000, the da Vinci^®^ surgical system has remained the leading platform in robot-assisted surgery. Although the da Vinci^®^ surgical system enables smooth operations by skilled surgeons, and it has a long clinical track record, introducing new platforms can bring competition and continuous technological advancements. In addition to widespread adoption, new robot-assisted surgical platforms have emerged, introducing novel features such as open-console designs, modularity, compatibility with conventional surgical instruments, a reduced footprint, and improved cost-effectiveness [13,14,15].

The HINOTORI™ surgical system, the first Japanese robot-assisted surgical platform, was introduced by Medicaroid Corporation (Kobe, Hyogo, Japan), Ltd. in 2020 [16]. Since its introduction, it has been adopted in various urologic surgical procedures. In 2020, Hinata et al. performed the first HINOTORI™-assisted radical prostatectomy for prostate cancer [2]. In 2023, Miyake et al. reported on HINOTORI™-assisted partial nephrectomy [17]. Furthermore, a systematic review and meta-analysis on HINOTORI™-assisted radical prostatectomy has been conducted, demonstrating perioperative and oncological outcomes comparable to those of existing robotic platforms [18].

The system is also contributing to the expansion of robot-assisted surgery in Japan. In 2023, Togami et al. reported on the use of HINOTORI™ for hysterectomy in gynecology [19]. In general surgery, Miura et al. documented its use in rectal cancer surgery [20], while Miyo et al. reported on its application in right hemicolectomy [21]. In 2024, Inoue et al. published findings on gastrectomy for gastric cancer [22], and Suda et al. reported its use in lung cancer surgery [23].

Only a small preliminary study has been published on the use of HINOTORI™ for sacrocolpopexy. In the study, Ichino et al. reported that although operative time was significantly shorter with the da Vinci^®^ system, no significant differences were observed between the two platforms regarding de novo SUI, recurrence rates, Overactive Bladder Symptom Score (OABSS), or urodynamic test results [24].

This study compared the surgical outcomes of RSC performed using the HINOTORI™ and da Vinci^®^ systems by a single skilled surgeon with uniformed surgical procedure. The outcomes observed in this cohort may have been influenced by the performance characteristics of the respective platforms. The h-RSC group had a slightly longer operative time (prolong: mean 9 min *p* < 0.005) and robotic console time (prolong: mean 14 min *p* < 0.001) compared to the d-RSC group. These differences were statistically significant. Analysis of the individual surgical steps showed no significant difference in dissection time between the two groups. This may be explained by the fact that the HINOTORI™ system is equipped with eight axes of motion—one more than the seven-axis configuration of the da Vinci^®^ system. Its design eliminates the need for trocar docking and reduces arm-to-arm collision, which may contribute to comparable performance in dissection. Additionally, the EndoWrist instruments used for dissection appear to function similarly between the two platforms. However, suturing time was significantly longer in the h-RSC group. This difference may be attributed to the fact that, in the d-RSC group, the surgeon performed suture cutting using an integrated tool, whereas in the h-RSC group, an assistant manually cut the sutures. Additionally, the difference in the rotational axis of the needle driver may have contributed to this disparity; the da Vinci^®^ system allows for 540 degrees of rotation, while the HINOTORI™ system is limited to 520 degrees. Future advancements in EndoWrist instrumentation may facilitate a reduction in operative time. However, these minor differences may not hold clinical significance. Furthermore, no significant differences were observed in perioperative complications, postoperative hospital stay, anti-incontinence surgery, or recurrence rates between the two groups. As this was a retrospective, single-center study, potential biases such as selection bias and unmeasured confounding factors cannot be completely excluded. Further well-designed, multicenter, prospective studies may be warranted to validate the clinical benefits of this novel platform in routine surgical practice.

### Limitations

This study has several limitations that should be acknowledged. First, this was a retrospective, relatively small-scale, single-center study with inherent methodological limitations, including the absence of a well-structured analytical framework. Second, due to the different initiation periods for using the two robotic platforms, the observation periods differed between the groups. Third, the relatively short follow-up duration limits the ability to assess long-term outcomes such as recurrence. Finally, multicenter prospective studies are needed to better evaluate functional outcomes and platform-specific performance.

## 5. Conclusions

HINOTORI™ maintains surgical precision comparable to da Vinci^®^. The HINOTORI™ surgical system can expand the options for robot-assisted surgery and contribute to the widespread adoption of surgical robotic systems.

## Figures and Tables

**Table 1:** Baseline Characteristics and Perioperative Outcomes Between the h-RSC and d-RSC Groups (Single Surgeon).

	h-RSC (*n* = 30)	d-RSC (*n* = 95)	*p*-Value
Baseline characteristics			
Median age, years (range)	75 (61–91)	75 (48–90)	0.57
Median BMI, kg/m^2^ (range)	23.3 (17.2–29.5)	23.9 (18.6–36.4)	0.33
Median parity, times (range)	2 (1–3)	2 (0–4)	0.60
POP-Q stage, *n* (%)			0.19
III	18 (60)	69 (72.6)	
IV	12 (40)	26 (27.4)	
Post hysterectomy, *n* (%)	7 (23.3)	37 (38.9)	0.06
With ventral rectopexy, *n* (%)	4 (13.3)	12 (12.6)	0.46
Perioperative outcomes			
operative times, min (range)	148 (121–200)	139 (98–215)	<0.005 *
robo time, min (range)	109 (84–160)	95 (59–156)	<0.001 *
postoperative length of stay, day (range)	6 (4–11)	5 (3–12)	0.96
perioperative complications, *n* (%) #	0 (0)	2 (2.1)	0.42
observation period (mon)	8 (1–16)	3 (1–74)	<0.001 *

Abbreviations: h-RASC, robot-assisted sacrocolpopexy with hinotori™ surgical system; d-RASC, robot-assisted sacrocolpopexy with da Vinci^®^ surgical system; BMI, body mass index; POP-Q, pelvic organ prolapse-quantification. #: bladder injury 2. * Statistically significant.

**Table 2:** Baseline Characteristics and Postoperative Outcomes Between the h-RSC and d-RSC Groups (Single Surgeon; ≥6-Month Follow-up).

	h-RSC (*n* = 22)	d-RSC (*n* = 89)	*p*-Value
Baseline characteristics			
Median age, years (range)	75 (61–91)	75 (48–90)	0.86
Median BMI, kg/m^2^ (range)	22.8 (17.2–29.5)	23.9 (18.6–36.4)	0.17
Median parity, times (range)	2 (1–3)	2 (0–4)	0.61
POP-Q stage, *n* (%)			0.12
III	12 (54.5)	64 (71.9)	
IV	10 (45.5)	25 (28.1)	
Post hysterectomy, *n* (%)	6 (27.3)	36 (40.4)	0.13
With ventral rectopexy, *n* (%)	4 (18.2)	9 (10.1)	0.29
Postoperative outcomes			
postoperative length of stay, day (range)	5 (4–8)	5 (3–12)	0.67
postoperative complications, *n* (%) ##	0 (0%)	2 (2.2%)	0.48
mesh erosion, *n* (%)	0 (0%)	0 (0%)	-
surgical recurrence, *n* (%)	1 (4.5%)	2 (2.2%)	0.55
mid-urethral sling surgery, *n* (%)	1 (4.5%)	9 (10.1%)	0.41
observation period (mon)	7 (6–16)	33 (9–74)	<0.001 *

Abbreviations: h-RASC, robot-assisted sacrocolpopexy with hinotori™ surgical system; d-RASC, robot-assisted sacrocolpopexy with da Vinci^®^ surgical system; BMI, body mass index; POP-Q, pelvic organ prolapse-quantification. ##: surgical site infection 2. * Statistically significant.

**Table 3:** Preoperative and postoperative PFDI-20 value in the h-RSC and d-RSC groups.

	h-RSC (*n* = 12)			d-RSC (*n* = 67)		
	Preoperative	Postoperative	*p*-Value	Preoperative	Postoperative	*p*-Value
PFDI-20, median (range)	96.4 (73–243.8)	19.9 (0–68.4)	<0.001 *	95.8 (37.5–245.9)	18.8 (0–108.3)	<0.001 *
POPDI-6, median (range)	41.7 (25–75)	0 (0–33.3)	<0.001 *	37.5 (8.3–91.7)	0 (0–45.8)	<0.001 *
CRADI-8, median (range)	17.2 (0–81.3)	6.3 (0–31.3)	0.10	15.6 (0–71.9)	3.1 (0–40.6)	<0.001 *
UDI-6. median (range)	37.5 (25–87.5)	6.25 (0–29.2)	<0.01 *	37.5 (4.2–104.2)	8.3 (0–62.5)	<0.001 *

Data reflect responses from participants who completed the PFDI-20 (HINOTORI™: *n* = 12; da Vinci^®^: *n* = 67). Abbreviations: h-RASC, robot-assisted sacrocolpopexy with hinotori™ surgical system; d-RASC, robot-assisted sacrocolpopexy with da Vinci^®^ surgical system; PFDI, the Pelvic Floor Distress Inventory; POPDI, Pelvic organ prolapse symptoms; CRADI, Colorectal-anal symptoms; UDI, Urinary symptoms. * Statistically significant.

## Data Availability

The data that support the findings of this study are available on request from the corresponding author. The data are not publicly available due to privacy or ethical restrictions.
